# Altered Detrusor Gap Junction Communications Induce Storage Symptoms in Bladder Inflammation: A Mouse Cyclophosphamide-Induced Model of Cystitis

**DOI:** 10.1371/journal.pone.0104216

**Published:** 2014-08-06

**Authors:** Takeshi Okinami, Masaaki Imamura, Nobuyuki Nishikawa, Hiromitsu Negoro, Yoshio Sugino, Koji Yoshimura, Akihiro Kanematsu, Hikaru Hashitani, Osamu Ogawa

**Affiliations:** 1 Department of Urology, Graduate School of Medicine, Kyoto University, Kyoto, Japan; 2 Department of Urology, Hyogo College of Medicine, Nishinomiya, Hyogo, Japan; 3 Department of Cell Physiology, Nagoya City University Graduate School of Medical Sciences, Nagoya, Japan; Louisiana State University Health Sciences center, United States of America

## Abstract

Lower urinary tract symptoms (LUTS) include storage, voiding and post-micturition symptoms, featuring many urological diseases. Storage symptoms are the most frequent among these and associated with overactive bladder and non-bacterial bladder inflammation such as interstitial cystitis/bladder pain syndrome (IC/BPS). Gap junction, a key regulator of hyperactive conditions in the bladder, has been reported to be involved in pathological bladder inflammation. Here we report involvement of gap junction in the etiology of storage symptoms in bladder inflammation. In this study, cyclophosphamide-induced cystitis was adapted as a model of bladder inflammation. Cyclophosphamide-treated mice showed typical storage symptoms including increased urinary frequency and reduced bladder capacity, with concurrent up-regulation of connexin 43 (GJA1), one of the major gap junction proteins in the bladder. In isometric tension study, bladder smooth muscle strips taken from the treated mice showed more pronounced spontaneous contraction than controls, which was attenuated by carbenoxolone, a gap junction inhibitor. In voiding behavior studies, the storage symptoms in the treated mice characterized by frequent voiding were alleviated by 18α-glycyrrhetinic acid, another gap junction inhibitor. These results demonstrate that cyclophosphamide-induced mouse model of cystitis shows clinical storage symptoms related with bladder inflammation and that gap junction in the bladder may be a key molecule of these storage symptoms. Therefore, gap junction in the bladder might be an alternative therapeutic target for storage symptoms in bladder inflammation.

## Introduction

Lower urinary tract symptoms (LUTS) are common in all populations, with a reported prevalence of approximately 60% [1]. Clinical management of LUTS is important because LUTS, although not life-threatening, can significantly diminish quality of life [1], [2]. LUTS consist of storage, voiding and post-micturition symptoms, with the storage symptoms being the most frequent among these [3], [4]. For the treatment of storage symptoms anti-muscarinic agents are effective [5], [6]. However, there are issues associated with these drugs including a high burden of economic cost and significant adverse effects including dry mouth and constipation [7], [8], [9].

The typical disease displaying storage symptoms as a specific clinical phenotype is overactive bladder (OAB). OAB is a hyperactive condition of the bladder defined as a complex symptom syndrome which includes urinary urgency, frequency and nocturia with no obvious pathology such as urinary tract infection, tumor or urolithiasis [3]. Because of the difficulty in excluding all pathological disorders, clinically diagnosed OAB could involve misdiagnosed diseases of non-bacterial bladder inflammation such as interstitial cystitis/bladder pain syndrome (IC/BPS). In support of this, several studies demonstrated the presence of inflammatory changes in bladders from patients with storage symptoms, as well as high frequency of storage symptoms in patients with diseases of bladder inflammation [10], [11], [12], indicating a close relationship between hyperactivity and inflammation in the bladder. Meanwhile, mechanisms underlying hyperactive bladder conditions are divided into neurogenic and non-neurogenic mechanisms [13]. Indeed, anti-muscarinic agents, which target neurogenic mechanisms, have been reported to be not effective for storage symptoms in diseases of bladder inflammation such as chronic pelvic pain syndrome and IC/BPS [14], [15]. These data suggest that bladder inflammation could contribute to non-neurogenic mechanisms causing storage symptoms, but not neurogenic ones.

Previous studies demonstrated that gap junction proteins in the urothelium or detrusor smooth muscle of bladders function as key regulators for non-neurogenic mechanisms of various hyperactive bladder conditions [16], [17], [18], [19]. However, the role of gap junctions is still unclear in bladder inflammation. Gap junction channels provide a cytoplasmic continuity between adjacent cells allowing the intercellular exchange of ions and metabolites [20]. Gap junctions consists of several connexins, and connexin 43 (GJA1) is one of the major components of gap junctions in bladders [16], [21]. Several studies indicating the relationship between inflammation and up-regulation of GJA1 in bladders strongly suggested that gap junction function was involved in pathological conditions underlying bladder inflammation [19], [22].

The purpose of this study was to elucidate the role of gap junctions, as non-neurogenic mechanisms of storage symptoms, in bladder inflammation. In this study, gap junction function was evaluated in a mouse cyclophosphamide-induced inflammatory bladder model. The mouse model displayed some features of storage symptoms including voiding behavior and increased spontaneous contraction of bladder smooth muscle strips concurrent with up-regulated gap junction formation in the bladder smooth muscle layer. In addition, inhibition of gap junction function in the mouse model attenuated spontaneous contraction and improved storage symptoms, indicating that gap junction function is a key regulator of storage symptoms in bladder inflammation, and thus could be a novel therapeutic target.

## Materials and Methods

### Ethics Statement

All animal experiments were approved by the Kyoto University and Nagoya City University animal experiment committees (Permit Number: Medkyo13332 and H24M-06), and all animals used in this study were treated according to the guidelines for animal experimentation of the experimental animal center of Kyoto University and Nagoya City University.

### Animals

Five-week-old female Balbc/A mice were purchased from CLEA Japan (Tokyo, Japan) or Japan SLC, Inc. (Hamamatsu, Japan). Mice were housed at a constant room temperature with a cycle of 12-hours light (9∶00 am to 9∶00 pm) and 12-hours dark (9∶00 pm to 9∶00 am). Food and water were available *ad libitum*.

### Reagents

Cyclophosphamide (CYP) was purchased from LKT laboratories, Inc., (St. Paul, MN, USA). Corn oil, 18α-glycyrrhetinic acid (GA) and carbenoxolone disodium salt (CBX) were purchased from Sigma-Aldrich (St. Louis, MO, USA). Bay K8644 was purchased from Wako Pure Chemical Industries, Ltd. (Osaka, Japan).

### Mouse model of CYP-induced cystitis

Mice were injected intraperitoneally with 150 mg/kg body weight of CYP diluted in 15 ml/kg body weight of saline. For the inhibition of gap junction function study, mice were injected subcutaneously with 30 mg/kg body weight of 18α-GA diluted in 2.5 ml/kg body weight of corn oil 24 hours prior to CYP administration. Mice injected with vehicle alone, saline or corn oil respectively, served as controls.

### Tissue preparation

Mice treated with CYP were killed by cervical dislocation 0, 6, 24, 48 or 96 hours after CYP administration and the bladders were harvested. For the evaluation of GJA1 localization, the bladders were manually dissected to two layers; urothelium with suburothelium, and smooth muscle layer. The numbers of mice used for each experiment are 3 or 4 in each group.

### Micturition analysis for mice

The aVSOP (automated Voided Stain On Paper) method was performed to evaluate voiding behavior as described previously [17]. Mice were kept in a cage located over the recording equipment for voided urine. Mice had free access to food and water during the test. The recording equipment included rolled laminated filter paper which was pre-treated to turn the edge of urine stains deep purple and wound up under a water-repellent wire lattice. The micturition assessment machines for the aVSOP method were manufactured by Real-designs Co. Ltd. (Kyoto, Japan). Voiding behavior was analyzed under 12 hours light and 12 hours dark conditions from two days prior to CYP administration. The area and time of stains were recorded and voided volume was calculated from each stain using a standard curve of the stained area of murine urine volumes ranging from 5 to 600 µl. Maximum voided volume was calculated to select the largest area per 12 hours light or dark period for each mouse. The numbers of mice used for each experiment are 3 or 4 with or without CYP respectively, and 5 or 6 in CYP-induced cystitis with or without 18α-GA respectively.

### RNA extraction

Total RNA was extracted from bladder tissues with RNeasy Mini kits (Qiagen, Hilden, Germany) according to the manufacturer's protocols.

### Affymetrix GeneChip array

Comprehensive gene expression analysis was performed for total RNA isolated from the bladders of mice with CYP-induced cystitis or control mice (n = 3 for each) 48 hours after treatment with the GeneChip Mouse Gene 1.0 ST Array containing probes for more than 26000 well-annotated genes. Aliquots of 100 ng total RNA were used for each sample. Target labeling, array hybridization, washing and staining were performed as described in the GeneChip Whole Transcript Sense Target Labeling manual (http://www.affymetrix.com). Arrays were scanned using the GeneChip Scanner 3000 7G (Affymetrix, Santa Clara, CA, USA). Data acquisition was performed with the Affymetrix GeneChip Command Console Software. Data analysis was performed with GeneSpring GX Version 11.5.1 (Agilent Technologies, Santa Clara, CA, USA). Of a total of 28853 genes on the microarray, genes with more than 80% signal intensity (23701 genes) were used for comprehensive analysis. Differences in expression between the CYP-induced cystitis group and the control group were calculated. Unpaired t test was performed for the microarray analysis.

### Real-time quantitative RT-PCR (qPCR)

cDNA was synthesized from total RNA using ReverTra Ace qPCR RT Kit (TOYOBO, Osaka, Japan). qPCR was performed with SYBR Green PCR Master Mix (Life Technologies, Carlsbad, CA, USA) and a 7300 Real-time PCR system (Life Technologies). The thermal cycling conditions were 94°C for 15 s, 60°C for 15 s, and 72°C for 1 min. Values were adjusted relative to the expression levels of the housekeeping gene *Gapdh*. Primer sequences are shown in Table 1. The ΔΔCt method was used to determine the relative gene expression of the genes of interest.

**Table 1:** The sequences of primers used for real-time quantitative PCR.

Gene symbol		Primer sequences	Product length (bp)
*Gja1*	Forward	5′-CCA TCC AAA GAC TGC GGA T-3′	138
	Reverse	5′-GTA ATT GCG GCA CGA GGA A-3′	
*Cox2*	Forward	5′-TGG GTG TGA AGG GAA ATA AGG-3′	104
	Reverse	5′-CAT CAT ATT TGA GCC TTG GGG-3′	
*Nos2*	Forward	5′-TCA GCC AAG CCC TCA CCT AC-3′	108
	Reverse	5′-CCA ATC TCT GCC TAT CCG TCT C-3′	
*Ptger4*	Forward	5′-GTG GTG CTC ATC TGC TCC ATT C-3′	102
	Reverse	5′-CTG CAA ATC TGG GTT TCT GCT G-3′	
*Ngf*	Forward	5′-AAT AGC TGC CCG AGT GAC AG-3′	124
	Reverse	5′-GTC TGA AGA GGT GGG TGG AG-3′	
*Trpv1*	Forward	5′-TGT GGA GGT GGC AGA TAA CA-3′	100
	Reverse	5′-CTT CAG TGT GGG GTG GAG TT-3′	
*Chrm2*	Forward	5′-CTG GAG CAC AAC AAG ATC CAG AAT-3′	70
	Reverse	5′-CCC CCT GAA CGC AGT TTT CAG T-3′	
*Chrm3*	Forward	5′-GCA AGA CCT CTG ACA CCA ACT-3′	92
	Reverse	5′-AGC AAA CCT CTT AGC CAG CG-3′	
*Chrm4*	Forward	5′-CGG CTA CTG GCT CTG CTA CGT CAA-3′	122
	Reverse	5′-CTG TGC CGA TGT TCC GAT ACT GG-3′	
*Gapdh*	Forward	5′-GCA CAG TCA AGG CCG AGA AT-3′	131
	Reverse	5′-GCC TTC TCC ATG GTG GTG AA-3′	

### Immunoblotting

Whole bladders were lysed with buffer containing 50 mM Tris-HCl (pH 7.4), 0.1% SDS, 1% Nonidet P-40 (octyl phenoxypolyethoxylethanol), 0.5% sodium deoxycholate, 150 mM sodium chloride, 1 mM sodium fluoride and 1 mM sodium vanadate supplemented with protease inhibitor cocktail (Nacalai Tesque, Kyoto, Japan). Total protein concentrations were determined using the DC protein assay (Bio-Rad Laboratories, Richmond, CA, USA). Protein lysates (20 µg) were resolved by sodium dodecylsulfate polyacrylamide gel electrophoresis and transferred to polyvinylidene difluoride membranes (Millipore, Bedford, MA, USA) using a Mini Trans-Blot Cell system (Bio-Rad Laboratories). Membranes were blocked with 1% bovine serum albumin diluted in TBST (BSA/TBST) and incubated with primary antibodies diluted in 1% BSA/TBST followed by incubation with horseradish peroxidase-conjugated secondary antibodies diluted in 1% BSA/TBST and developed for reading by enhanced chemiluminescence (SuperSignal West Pico Chemiluminescent Substrate, Thermo Scientific, Rockford, IL, USA). Images were acquired with the LAS-4000 imaging system (Fujifilm Life Science, Tokyo, Japan). Anti-connexin 43 (GJA1, C6219, Sigma-Aldrich, 1∶10000), anti-cytokeratin 18 (KRT18, 04-586, Millipore, Billerica, MA, USA, 1∶5000), anti-alpha smooth muscle actin (ACTA2, ab5694, Abcam, Cambridge, England, 1∶10000) and anti-GAPDH (GAPDH, 2118, Cell Signaling Technology, Danvers, MA, USA, 1∶5000) were used as the primary antibodies.

### Transmission electron microscopy (TEM)

Whole bladders were fixed with 4% paraformaldehyde and 2% glutaraldehyde in 0.1 M phosphate buffer for 24 hours at 4°C. After washing in 0.1 M phosphate buffer, the samples were post-fixed with 1% OsO_4_ in 0.1 M phosphate buffer for 2 hours. After dehydration with graded concentrations of ethanol and propylene oxide, the samples were penetrated and polymerized with Luveak 812 (Nacalai Tesque, Kyoto, Japan). Ultrathin 80 nm sections were cut with a microtome EM UC6 (Leica, Hiederberg, Germany), placed on mesh copper grids, stained with uranyl acetate and lead citrate, and examined at 80 kV acceleration voltage using an H7650 electron microscope (Hitachi, Tokyo, Japan).

### Measurement of isometric tension

The bladder was pinned down in a dissecting dish with the urothelial side facing up. The urothelial layer was then dissected away with ophthalmology scissors, leaving the detrusor smooth muscle layer for use in the study. Silk threads were tied around both ends of the smooth muscle strips (8–10 mm long; 1–3 mm wide). Preparations were transferred to 2 ml organ baths and superfused with warmed (35°C) physiologic salt solution (PSS) at a constant flow rate (2–3 ml/min). The composition of PSS was 137.5 mM Na^+^, 5.9 mM K^+^, 2.5 mM Ca^2+^, 1.2 mM Mg^2+^, 15.5 mM HCO3^−^, 1.2 mM H_2_PO_4_^−^, 134 mM Cl^−^ and 11.5 mM glucose. The pH of PSS was 7.2 when bubbled with 95% O_2_ and 5% CO_2_, and the measured pH of the organ bath solution was approximately 7.4. One thread was fixed to the bottom of the organ bath, while the other was connected to an isometric force transducer connected to a bridge amplifier. Changes in isometric tension were digitized using a Digidata 1440A interface (Axon Instruments, Inc., Foster City, CA, USA). After 30 minutes of incubation with warmed PSS, an initial tension of appropriately 1 mN was applied to each preparation. Preparations were then equilibrated for another 30–60 minutes. Increasing concentrations of Bay K8644, an L-type Ca^2+^ channel opener, were cumulatively added to the solution. The muscle strip was incubated for at least 30 minutes at each concentration, and data from only the last 10 minutes of each period was analyzed. In the presence of 300 nM Bay K8644, 30 µM CBX, a gap junction blocker, was added to the solution and muscle strip was incubated for at least 20 minutes.

### Statistical analysis

All data are expressed as the mean ± SD. GraphPad Prism 4.0 (GraphPad Software, Inc., La Jolla, CA, USA) was used for statistical analysis. Unpaired or paired t test, Dunnett's multiple comparison test and two-way repeated measure ANOVA were performed when appropriate. *P*<0.05 was regarded as statistically significant.

## Results

### CYP-induced cystitis mouse model showed reduced bladder capacity and increased urinary frequency

**Figure 1:**
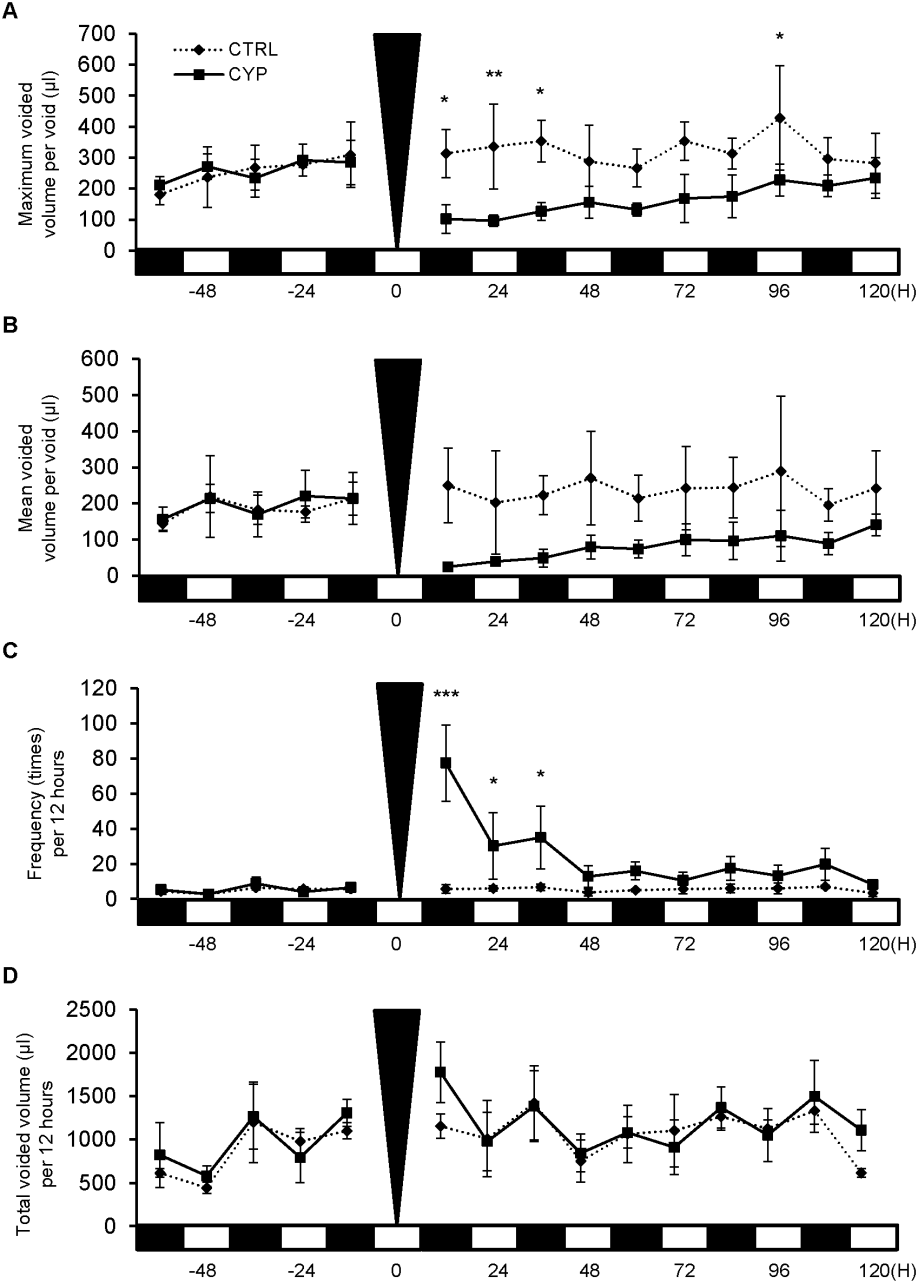
Voiding behavior of mice with cystitis. Micturition patterns were analyzed in the CYP-induced cystitis group (CYP; n = 3) and the sham-treated control group (CTRL; n = 4) with the aVSOP method. *A*: Maximum voided volume per void. The CYP group demonstrated a significant decrease in volume at 12, 24, 36 and 96 hours when compared to the control group. *B*: Mean voided volume per void. The CYP group demonstrated a decrease in volume. *C*: Urinary frequency per 12 hours. The CYP group displayed a significant increase in frequency at 12, 24 and 36 hours. *D*: Total urine volume per 12 hours. There was no change in both groups. The black triangle shows the time of CYP administration. On the X-axis, the black squares indicate dark periods (9∶00 pm to 9∶00 am) and the white squares indicate light periods (9∶00 am to 9∶00 pm). Two way repeated measure ANOVA and Bonferroni post-test were performed and *P*<0.05 was regarded as significant. *P<0.05, **P<0.01, ***P<0.001.

aVSOP analysis data showed that the maximum voided volume per void, which reflect bladder capacity, was significantly decreased in the CYP-treated group when compared to the control group (Fig. 1A, *P*<0.05, CTRL, n = 4; CYP, n = 3). Mean voided volume per void was decreased in the CYP-treated group (Fig. 1B). Urinary frequency was also significantly increased in the CYP-induced cystitis group (Fig. 1C, *P*<0.05, CTRL, n = 4; CYP, n = 3), however there was no change in the total voided volume (Fig. 1D).

### Mice with CYP-induced cystitis showed elevated connexin 43 expression in the bladder, independently of other OAB-related genes

**Figure 2:**
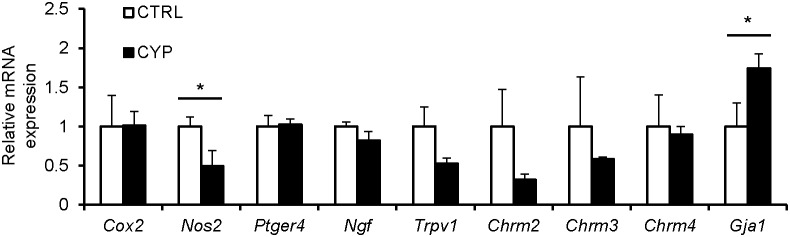
Expression of OAB-related genes in bladders of cystitis mice. Levels of *Gja1* or other known OAB-related genes were analyzed in the CYP-induced cystitis group (CYP; n = 3) and the sham-treated control group (CTRL; n = 3) by qPCR. The CYP group demonstrated a significant increase in *Gja1* mRNA expression, but no change or decrease in the expression of other genes, when compared to the control group. Unpaired t tests were performed and *P*<0.05 was regarded as statistically significant; *P<0.05.

Microarray results showed that expression of *Gja1*, which we previously proposed as the key gene related to bladder function [16], [17], [18], was elevated 1.40 fold in the bladders of CYP-treated mice. The microarray data discussed in this publication have been deposited in NCBI's Gene Expression Omnibus (Edgar et al., 2002) and are accessible through GEO Series accession number GSE55986 (http://www.ncbi.nlm.nih.gov/geo/query/acc.cgi?acc=GSE55986). To validate microarray results, the expression of *Gja1* and other known OAB-related genes was evaluated in whole bladders by qPCR. The data showed that *Gja1* mRNA expression in bladders of the CYP-treated group was significantly up-regulated (*P*<0.05, n = 3 for each group), but that the expression of other genes was not changed or was down-regulated when compared to the control group (Fig. 2).

### Up-regulated gap junction formation was observed in the bladder smooth muscle layer of the mice with CYP-induced cystitis

**Figure 3:**
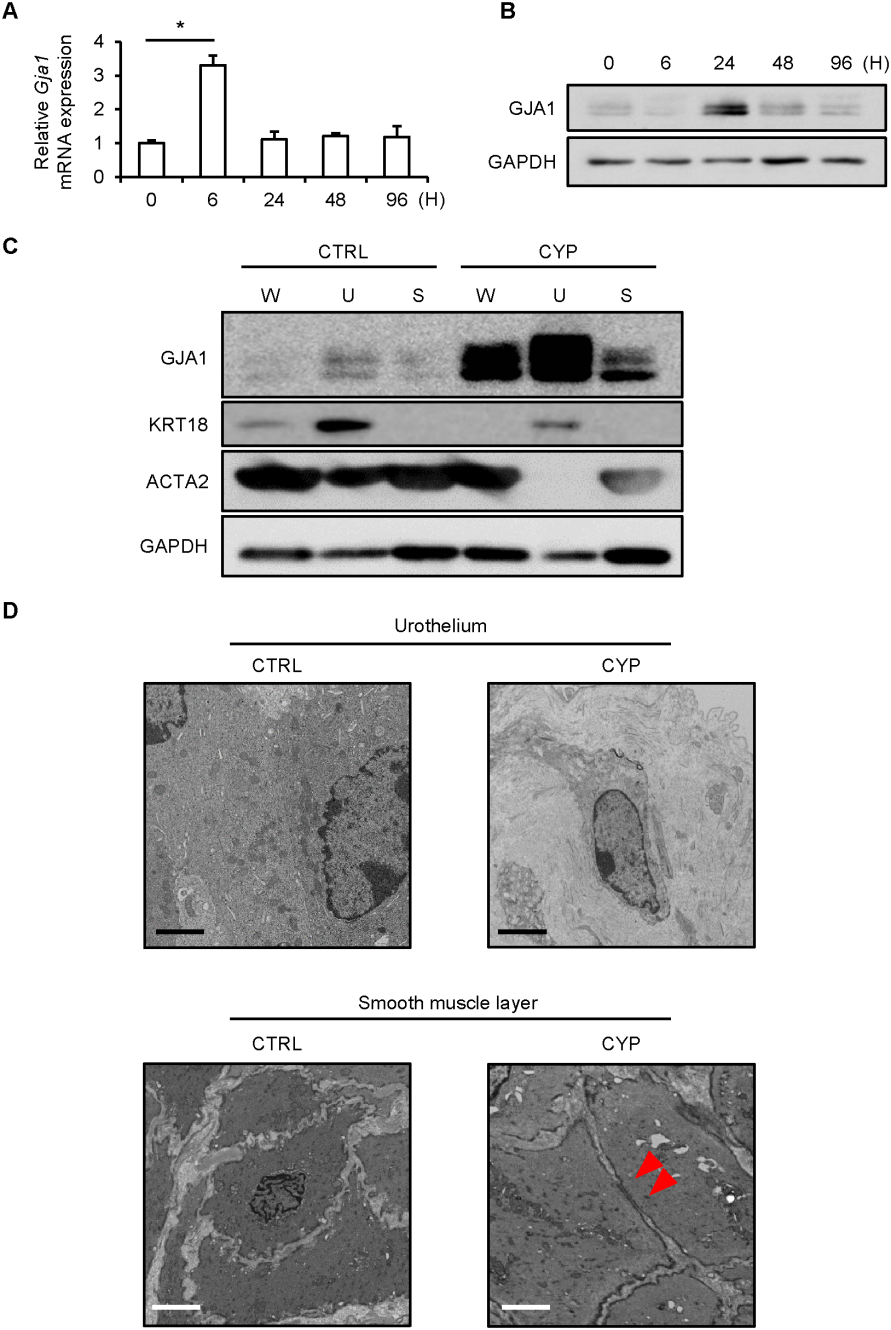
GJA1 expression and gap junction formation in the bladders of mice with cystitis. *A*: qPCR revealed significantly elevated expression of *Gja1* mRNA in the whole bladder at 6 hours after treatment (n = 4). Dunnett's multiple comparison test was performed. *P*<0.05 was regarded as statistically significant; *P<0.05. *B*: Immunoblotting data revealed up-regulation of GJA1 protein in the whole bladder at 24 hours after treatment (n = 3). The representative data shown were consistently replicated in other experiments. *C*: Immunoblotting data revealed that GJA1 expression was stronger in the urothelium with suburothelium (U) than in the smooth muscle layer (S) in the CYP-induced cystitis group (n = 3). The representative data shown were consistently replicated in other experiments. W: the whole bladder. *D*: TEM study revealed that urothelial cells were attached with each other in the sham-treated control group (CTRL; n = 3) but disconnected in the CYP-induced cystitis group (CYP; n = 3). Gap junctions were not observed in either group. In contrast, gap junctions were not found in the smooth muscle layer of the CTRL group but appeared in that of the CYP group. The representative data shown were consistently replicated in other experiments. Red arrow heads indicate gap junction. Scale bars indicate 2 µm.

qPCR results showed that expression of *Gja1* mRNA increased significantly 6 hours after CYP administration (Fig. 3A). Furthermore, immunoblotting results showed that expression of GJA1 protein was up-regulated 24 hours after CYP administration (Fig. 3B). Immunoblotting analysis showed that GJA1 protein was expressed in both urothelium and bladder smooth muscle layer and expression level in urothelium was relatively stronger than that in bladder smooth muscle layer (Fig. 3C). Meanwhile, TEM study for the mouse bladders with CYP-induced cystitis showed that urothelial cells existed separately from each adjacent cell with no gap junction formation but up-regulated gap junction formation was observed in the smooth muscle layer (Fig. 3D). The histological findings showed that urothelial cells in the control mice were connected with each other but those in the model mice were separately distributed in the presence of abundant intercellular substance (fig. S1), confirming TEM study data.

### Mice with CYP-induced cystitis showed spontaneous contraction in the bladder mediated through gap junction function

**Figure 4:**
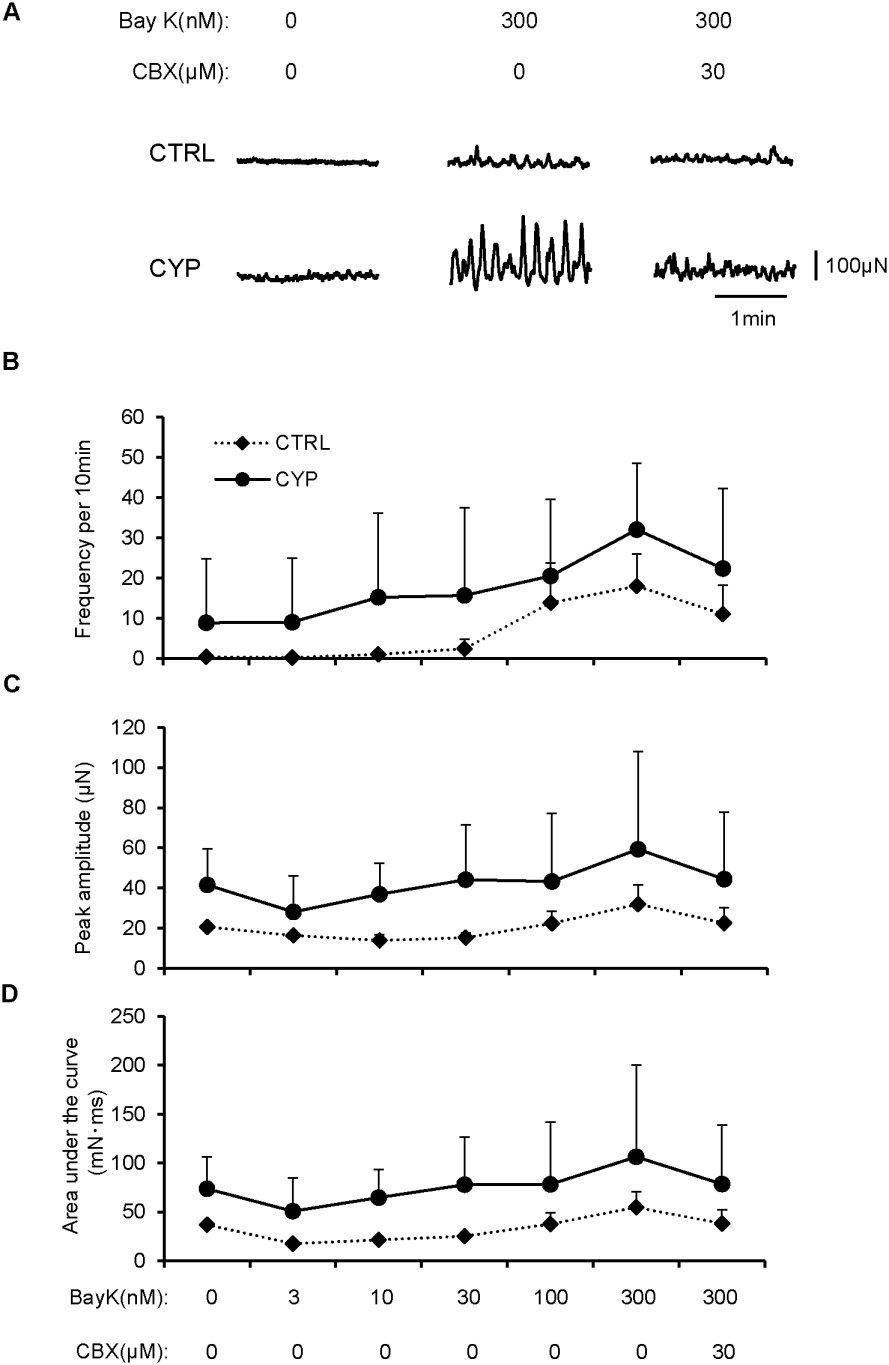
Analysis of spontaneous contraction in mice with cystitis. Isometric tension measurement of bladder smooth muscle strips from the CYP-induced cystitis group (CYP; n = 6) and the sham-treated control group (CTRL; n = 5) were performed with or without Bay K8644 (Bay K) or carbenoxolone (CBX). *A*: Representative data revealed that spontaneous contraction and its enhancement by Bay K8644 were observed in the CYP group. Bay K8644-induced spontaneous contraction was blocked by CBX in the CYP group. *B–D*: Evaluation of parameters of spontaneous contraction. Frequency per 10 minutes (B), peak amplitude (C) and area under the curve (D) of spontaneous contraction increased in the CYP group when compared to the control group. CBX down-regulated increased levels of each parameter of spontaneous contraction in the CYP group (B–D).

Since GJA1 is one of the major components of the gap junction channel in the bladder [16], [21], GJA1 up-regulation may result in enhanced gap junction-mediated signaling. To evaluate if GJA1 up-regulation was associated with altered detrusor smooth muscle contractility, properties of spontaneous contraction in the bladders of cystitis model mice were compared with sham-operated mice. Consistent with previous studies, mouse detrusor muscle strips often failed to exhibit measurable spontaneous contraction, presumably due to relatively low intercellular coupling [23], [24]. Therefore, spontaneous contraction was induced by Bay K8644, an L-type Ca^2+^-channel activator, which increased spontaneous action potentials and corresponding Ca^2+^ transients (fig. S2). Smooth muscle strips taken from bladders of sham-operated mice did not exhibit spontaneous contraction (n = 5), however increasing concentrations of Bay K8644 induced spontaneous contraction (Fig. 4A). Muscle strips taken from bladders of two of six (33.3%) CYP-treated mice developed spontaneous contraction spontaneously, while bladders of the remaining four CYP-treated mice were quiescent (Fig. 4A). Increasing concentrations of Bay K8644 enhanced or induced spontaneous contraction, and analysis of spontaneous contraction revealed increases in the frequency, amplitude and area under the curve of spontaneous contraction in CYP-treated mice in comparison to sham-operated mice (Fig. 4B–D, CTRL, n = 5; CYP, n = 6). The enhanced spontaneous contraction induced by Bay K8644 was attenuated by the gap junction blocker CBX (Fig. 4A–D).

### Gap junction function mediated changes in voiding behavior in mice with CYP-induced cystitis

**Figure 5:**
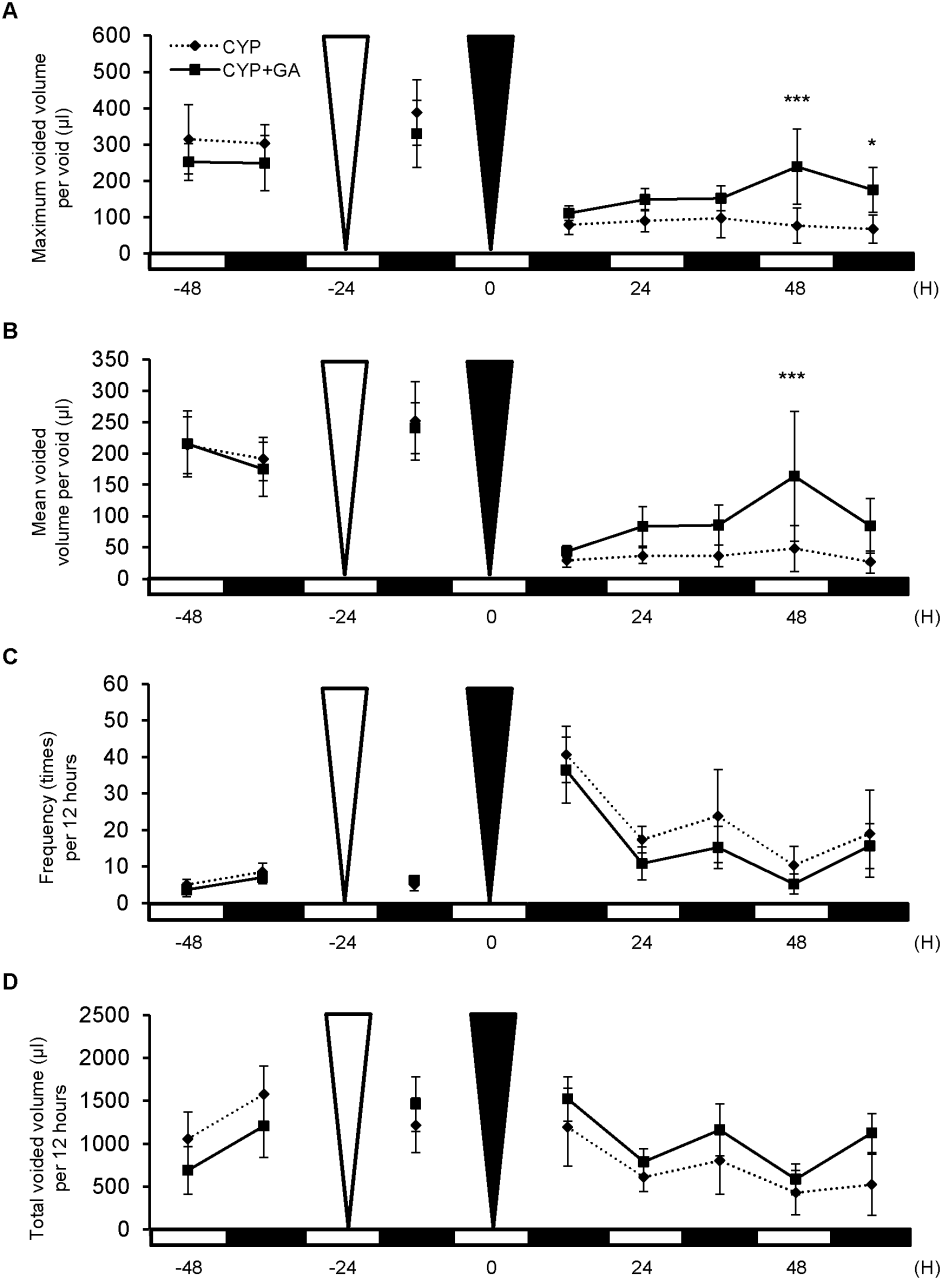
Voiding behavior in mice with cystitis treated with 18α-glycyrrhetinic acid (GA). Micturition patterns were analyzed in the CYP-induced cystitis group in the presence (CYP+GA; n = 5) or absence (as control, CYP; n = 6) of 18α-GA with the aVSOP method. *A*: Maximum voided volume per void. The CYP+GA group demonstrated a significantly increased volume at 48 and 60 hours when compared to the control group. *B*: Mean voided volume per void. The CYP+GA group revealed a significant increase in volume at 48 hours. *C*: Urinary frequency per 12 hours. The CYP+GA group revealed lower frequency at each period examined. *D*: Total urine volume per 12 hour. The CYP+GA group revealed greater volume at each period. The white and black triangles show the time of 18α-GA and CYP administration, respectively. On the X-axis, the black squares indicate dark periods (9∶00 pm to 9∶00 am) and the white squares indicate light periods (9∶00 am to 9∶00 pm). Two way repeated measure ANOVA and Bonferroni post-test were performed. *P*<0.05 was regarded as statistically significant; *P<0.05, ***P<0.001.

To evaluate the involvement of gap junction function on voiding behavior in CYP-induced cystitis, aVSOP analysis was performed which included treatment of mice with cystitis with 18α-GA, a gap junction inhibitor. The group treated with 18α-GA showed a significant increase in maximum and mean voided volume per void when compared to the sham-treated group (Fig. 5A, B). The group treated with 18α-GA also showed a decrease in urinary frequency and increase in total voided volume (Fig. 5C, D). 18α-GA has been reported to uncouple gap junction [25]. To confirm whether 18α-GA inhibited gap junction in such a functional manner or changed protein expression itself, immunoblotting for GJA1 in each model was performed. The results showed that 18α-GA treatment did not alter GJA1 protein expression (fig. S3).

## Discussion

In this study, we have demonstrated that: 1) the CYP-induced model of cystitis showed features of storage symptoms, 2) gap junction formation was up-regulated in the bladder smooth muscle layer of the model mice, 3) spontaneous contraction was enhanced in detrusor smooth muscles taken from the treated mice, 4) gap junction blockade alleviated storage symptoms in the model mice. Therefore, gap junction function could regulate detrusor activity in the CYP-induced mouse model of cystitis.

A major finding of this study is that altered gap junction function results in functional changes in bladder inflammation as storage symptoms. Previous studies showed that inflammatory stimuli regulated gap junction protein expression but did not induce any functional changes *in vivo* [26], [27]. Therefore, it was unknown whether changes in the expression of gap junction proteins actually may be associated with function in bladder inflammation. In this study, we revealed that GJA1 up-regulation resulted in changes in voiding behavior of the cystitis model mice through gap junction formation in the smooth muscle layer, indicating that molecular changes in bladder inflammation affected bladder storage function. It is well-known that changes in cardiac GJA1 expression induce arrhythmia, which is evidence of major functional changes in the heart [28], [29]. This phenomenon supports our data that molecular changes in gap junction proteins affect functional properties of the bladder.

Consistent with the above finding, spontaneous contraction in isometric tension studies appears to be enhanced in the bladder of the CYP treated mice. In muscle strips from the model mice, spontaneous contraction was observed even without any stimulus, and Bay K8644 induced more pronounced spontaneous contraction in comparison with those from sham-operated mouse bladders. These findings suggested that spontaneous contraction could be a factor underlying the pathophysiology of bladder inflammation. Although spontaneous action potential and associated Ca^2+^ transient could be generated by single detrusor smooth muscle cells, the generation of spontaneous contraction requires synchrony amongst smooth muscle syncytium mediated by intercellular coupling through gap junctions [24], [30], [31], [32]. Bay K8644 appears to facilitate not only action potential generation in individual smooth muscle cells but also intercellular propagation of action potentials by increasing the open probability of L-type Ca^2+^ channels. Increased spontaneous contraction may mechanically stimulate afferent nerve endings to cause urinary urgency with detrusor hyperactivity [33]. Taking those findings into account, our model could reflect enhanced intercellular communication in detrusor muscles. Attenuation of spontaneous contraction with gap junction blockade by CBX also supports this notion.

Here we showed that our model had features of myogenic mechanisms that induced storage symptoms, but did not show up-regulation of genes associated with neurogenic mechanisms. Meanwhile, previous studies have implicated that neurogenic mechanisms were involved in bladder inflammation and caused functional changes [34], [35]. Differences between species might explain this discrepancy because mice were used in our model while rats were used in other studies. In addition, there is a possibility that gene expression involved in neurogenic mechanisms might be observed at other time points, because evaluation for gene expression in this model was performed only at 48 hours after CYP administration. Therefore, involvement of neurogenic mechanisms cannot be excluded. Nevertheless, our data suggest that myogenic mechanisms are the main contributors to functional change for the following reasons. First, spontaneous contraction was observed in muscle strips that had no extrinsic neuronal component. Second, blockade of gap junction function with CBX was effective in the contractile study as well as in *in vivo* voiding behavior measurement.

A limitation of this study is that the outcome of GJA1 up-regulation in the urothelium of the model mice is unclear. GJA1 was reported to be expressed in all layers of the bladder including urothelium [36], suburothelium [22], [37], [38] and smooth muscle [16], [17], [18],[19],[22]. In the urothelium, blockade of gap junctions attenuated ATP release from the urothelium [36]. In suburothelium, gap junctions are considered to form a network of interstitial cells and operates as a functional syncytium, integrating signals and responses [37]. Therefore, GJA1 up-regulation might activate those mechanisms in urothelium or suburothelium, contributing to the etiology of our cystitis model. Our speculation is that GJA1 up-regulation in the urothelium is induced by inflammation with compensation for lost cell-cell connection but does not lead to gap junction formation in such a short time course. There remains the possibility that GJA1 in the urothelium and/or suburothelium affects their functions *in vivo* in longer time course after inflammation. Further evaluation will be needed to confirm the possibility.

From the results of this study, this model may closely reflect patients who have clinical storage symptoms with bladder inflammation. There are some diseases that have storage symptoms concurrent with bladder inflammation, such as IC/BPS, chronic pelvic pain syndrome or cystitis induced by intravesical Bacillus Calmette-Guérin immunotherapy [14], [15], [39]. In such diseases of bladder inflammation, anti-muscarinic agents are not effective in the relief of storage symptoms [14], [15], [40]. Thus, new therapeutic treatments for these patients are required. 18α-GA has the advantage of maintaining contractile force, which is a major adverse effect of anti-muscarinic agents [16]. Therefore, 18α-GA or other gap junction inhibitors might be an alternative therapeutic option for storage symptoms in bladder inflammation.

## Conclusions

In this study, the CYP-induced model of cystitis closely reflects clinical storage symptoms in bladder inflammation. Gap junction function is regarded as a key regulator of the myogenic mechanisms inducing storage symptoms, and therefore its suppression might be an alternative therapeutic option for storage symptoms in bladder inflammation.

## Supporting Information

Figure S1 **Histological evaluation of CYP-induced cystitis.** Urothelial cells were arranged with connection to each other cells in the sham-treated control group (CTRL; n = 3) but separated with invasion of inflammatory cells in suburothelium in the CYP-induced cystitis group (CYP; n = 3). The representative data shown were consistently replicated in other experiments. Scale bars indicate 100 µm.
(TIF)

Figure S2 **Effects of Bay K8644 on spontaneous electrical and Ca^2+^ activity.** Intracellular recording demonstrated that Bay K8644 (100 nM) induced spontaneous action potentials (Ab) in preparations that did not previously exhibit action potentials (Aa) (n = 4). Fluo-4 Ca^2+^ imaging demonstrated that Bay K8644 (100 nM) increased the frequency of spontaneous Ca^2+^ transients (Ba and Bb) (n = 5).
(TIF)

Figure S3 **GJA1 expression with or without 18α-GA treatment.** Immunoblotting data revealed that there was no change in GJA1 protein expression in the whole bladders of mice with CYP-induced cystitis, with or without treatment with 18α-GA (n = 3 in each group). The representative data shown were consistently replicated in other experiments.
(TIF)
