# Supplementary figures and images for: Altered Detrusor Gap Junction Communications Induce Storage Symptoms in Bladder Inflammation: A Mouse Cyclophosphamide-Induced Model of Cystitis
Source: PLoS One. 2014 Aug 6;9(8):e104216. doi: 10.1371/journal.pone.0104216 (PMC4123906; doi:10.1371/journal.pone.0104216)

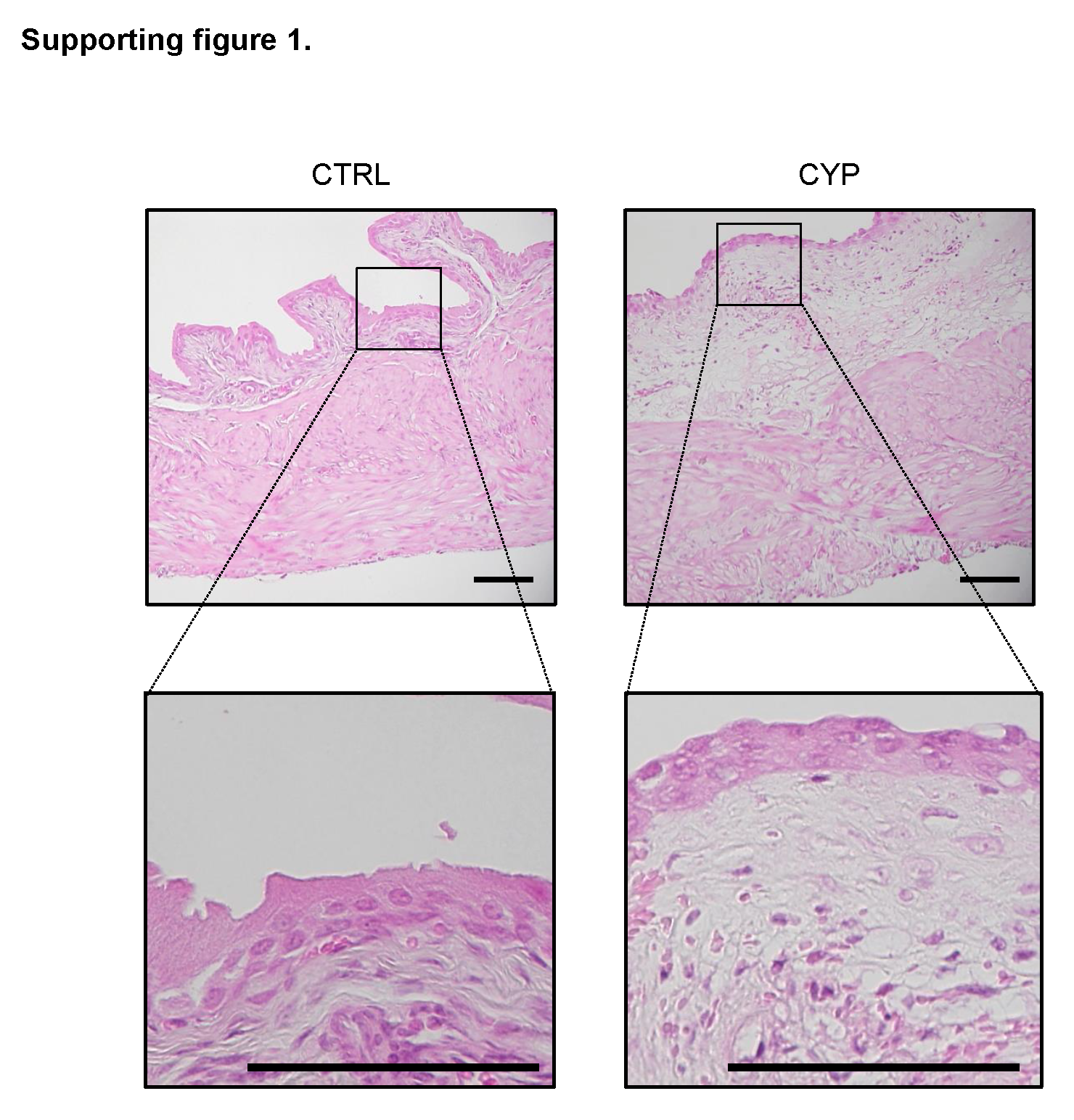

Supplement: Figure S1 — Histological evaluation of CYP-induced cystitis. Urothelial cells were arranged with connection to each other cells in the sham-treated control group (CTRL; n = 3) but separated with invasion of inflammatory cells in suburothelium in the CYP-induced cystitis group (CYP; n = 3). The representative data shown were consistently replicated in other experiments. Scale bars indicate 100 µm. (TIF) [file pone.0104216.s001.tif]

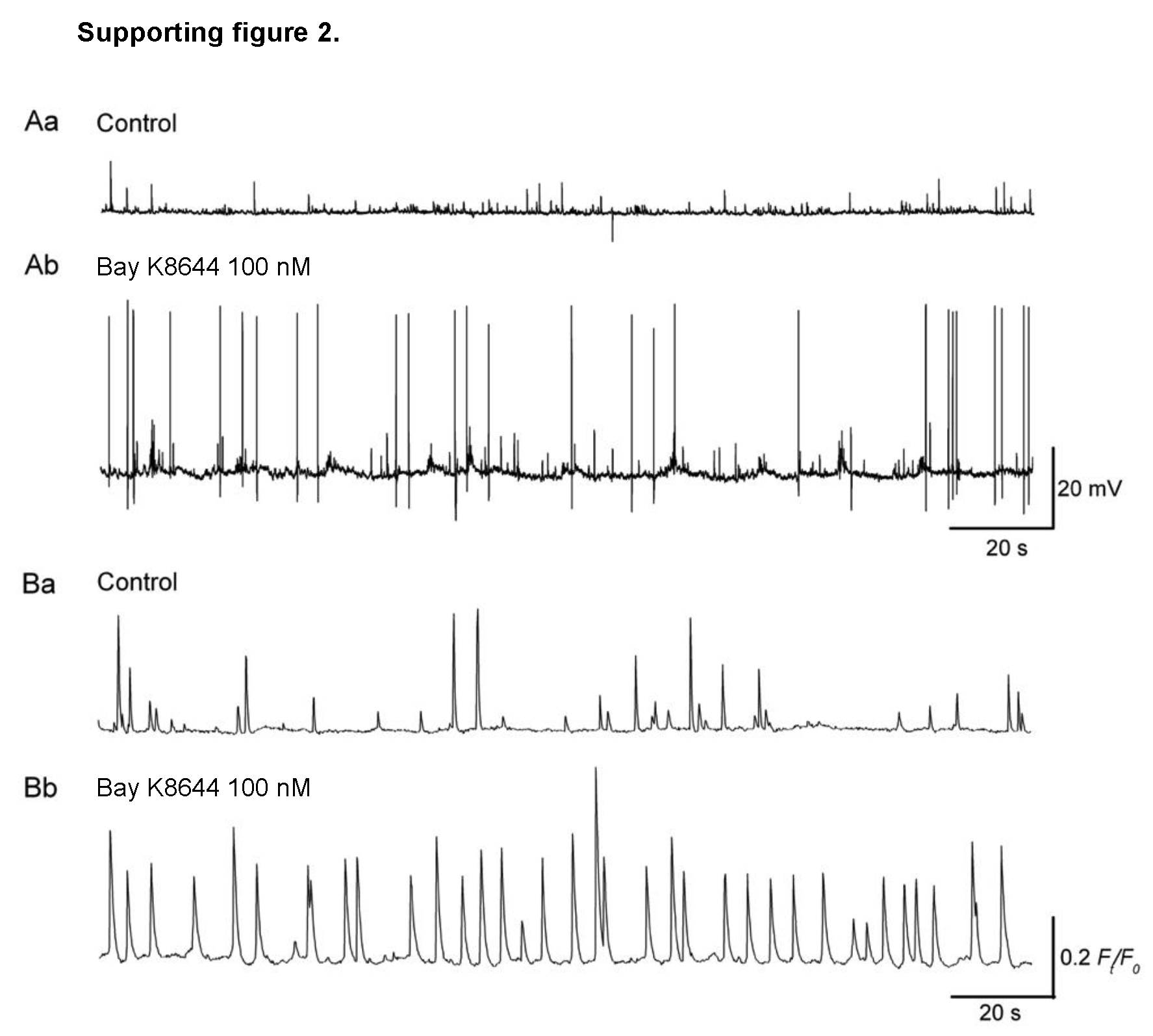

Supplement: Figure S2 — Effects of Bay K8644 on spontaneous electrical and Ca2+ activity. Intracellular recording demonstrated that Bay K8644 (100 nM) induced spontaneous action potentials (Ab) in preparations that did not previously exhibit action potentials (Aa) (n = 4). Fluo-4 Ca2+ imaging demonstrated that Bay K8644 (100 nM) increased the frequency of spontaneous Ca2+ transients (Ba and Bb) (n = 5). (TIF) [file pone.0104216.s002.tif]

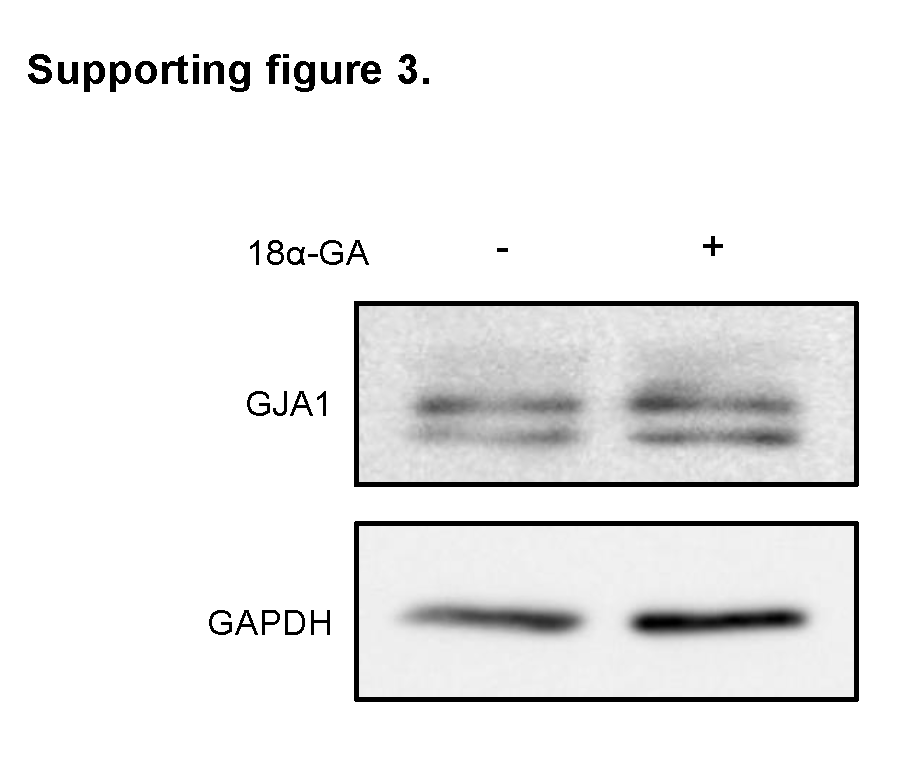

Supplement: Figure S3 — GJA1 expression with or without 18α-GA treatment. Immunoblotting data revealed that there was no change in GJA1 protein expression in the whole bladders of mice with CYP-induced cystitis, with or without treatment with 18α-GA (n = 3 in each group). The representative data shown were consistently replicated in other experiments. (TIF) [file pone.0104216.s003.tif]
